# Discovery of topological nodal-line fermionic phase in a magnetic material GdSbTe

**DOI:** 10.1038/s41598-018-31296-7

**Published:** 2018-09-05

**Authors:** M. Mofazzel Hosen, Gyanendra Dhakal, Klauss Dimitri, Pablo Maldonado, Alex Aperis, Firoza Kabir, Christopher Sims, Peter Riseborough, Peter M. Oppeneer, Dariusz Kaczorowski, Tomasz Durakiewicz, Madhab Neupane

**Affiliations:** 1 Department of Physics, University of Central Florida, Orlando, Florida 32816 USA; 2 Department of Physics and Astronomy, Uppsala University, P. O. Box 516, S-75120 Uppsala, Sweden; 3 Department of Physics, Temple University, Philadelphia, PA 19122 USA; 4 Institute of Low Temperature and Structure Research, Polish Academy of Sciences, 50-950 Wroclaw, Poland; 5 Condensed Matter and Magnet Science Group, Los Alamos National Laboratory, Los Alamos, NM 87545 USA; 6 Institute of Physics, Maria Curie - Sklodowska University, 20-031 Lublin, Poland

## Abstract

Topological Dirac semimetals with accidental band touching between conduction and valence bands protected by time reversal and inversion symmetry are at the frontier of modern condensed matter research. A majority of discovered topological semimetals are nonmagnetic and conserve time reversal symmetry. Here we report the experimental discovery of an antiferromagnetic topological nodal-line semimetallic state in GdSbTe using angle-resolved photoemission spectroscopy. Our systematic study reveals the detailed electronic structure of the paramagnetic state of antiferromagnetic GdSbTe. We observe the presence of multiple Fermi surface pockets including a diamond-shape, and small circular pockets around the zone center and high symmetry X points of the Brillouin zone (BZ), respectively. Furthermore, we observe the presence of a Dirac-like state at the X point of the BZ and the effect of magnetism along the nodal-line direction. Interestingly, our experimental data show a robust  Dirac-like state both below and above the magnetic transition temperature (*T*_*N*_  = 13 K). Having a relatively high transition temperature, GdSbTe provides an archetypical platform to study the interaction between magnetism and topological states of matter.

## Introduction

The discovery of three-dimensional (3D) topological insulators turned out to be an enthralling achievement of the last decade^1,2^. A 3D ℤ_2_ topological insulator (TI) is described as a crystalline solid that acts as a traditional insulator in the bulk but has surface Dirac electron states which are conducting, gapless, or even spin polarized^1–5^. The discovery of such materials with spin-momentum locking dates back to 2007^1–5^ when the bismuth-based 3D TI Bi_2_Se_3_ was reported. This initiated a domino effect of subsequent discoveries, where one new exotic state led to another, broadening our knowledge of quantum matter and increasing the number of researchers focused on this novel field. Consideration of electronic states protected by time-reversal, crystalline, and particle-hole symmetries have led to the prediction of many novel 3D materials, which can support Weyl, Dirac, and Majorana fermions, and new types of insulators such as topological crystalline insulators and topological Kondo insulators, topological superconductors, as well as 2D quantum spin Hall insulators with large band gaps capable of surviving room-temperature thermal excitations^3,6–11^. Recent attention has focused on exploring non-trivial topological behavior in metals and semimetals without bulk band gaps, which can support symmetry protected gapless points in the Brillouin zone^12–24^.

Topological materials offer transformational opportunities by providing platforms for entirely new classes of fundamental science studies of quantum matter as well as developing new paradigms for the design of devices for wide-ranging applications in next generation electronics, communications, and energy technologies^1,2^. It is envisaged that novel quantum materials will render another breakthrough in technology development. Up to date, available topological materials are mostly nonmagnetic, with very few magnetic topological semimetals^25–28^. Therefore, there is an especially urgent need to find new magnetic topological materials so that the unique potential for fundamental science studies and applications can be explored. This new realm may provide us with new exotic states which could be helpful in revolutionizing technology.

In this report, we present a systematic study of an antiferromagnetic compound GdSbTe in order to discover the topology and magnetism of this material using angle-resolved photoemission spectroscopy (ARPES), thermodynamic measurements, and first-principles calculations. Remarkably, GdSbTe is isostructural with the nodal-line semimetal ZrSiS^29,30^ and related compounds, which have been shown to exhibited a diamond-shaped Dirac line node as well as four-fold degenerate nodes at the high-symmetric points of the Brillouin zone (BZ)^29–41^. GdSbTe has a relatively high magnetic transition temperature *T*_*N*_ = 13 K, and hence its ordered state can be accessed by ARPES and other experimental techniques.

Our systematic electronic structure studies reveal the presence of multiple Fermi surface pockets such as a diamond-, and circular-shaped pockets around the Γ, and X points, respectively, in the first BZ of GdSbTe. Our data show that this system is not only structurally but also electronically similar to ZrSiS and related nonsymmorphic Dirac materials. Importantly, we observe a Dirac like state at the X point in both the paramagnetic and antiferromagnetic states. Furthermore, we reveal the effect of magnetism along the nodal-line direction of the BZ. Our findings provide a platform for discovering new exotic quantum phases emerging due to the interplay of magnetism with the topological phases.

## Results

### Crystal structure and sample characterization

**Figure 1:**
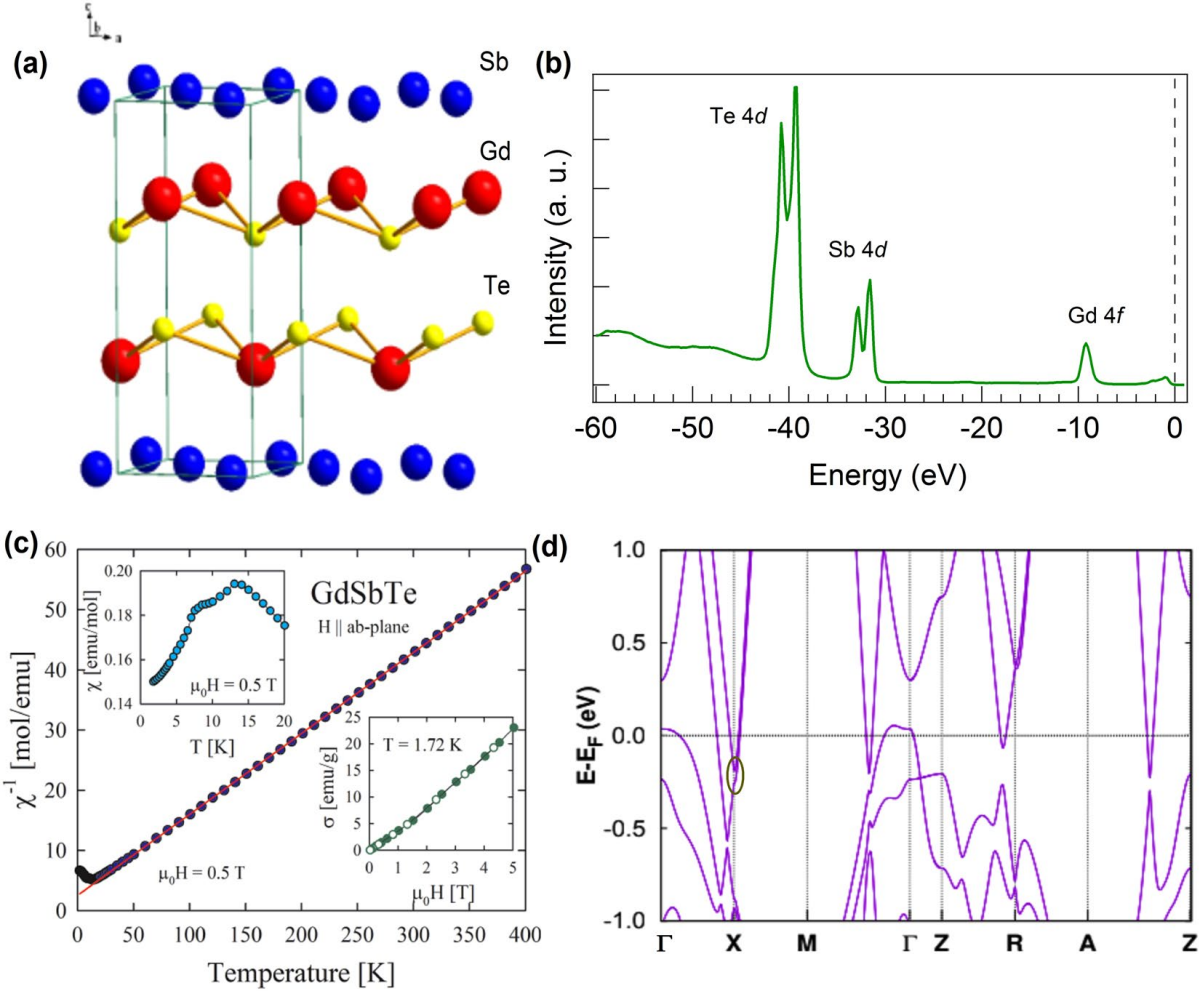
Crystal structure and sample characterization of GdSbTe. (**a**) Tetragonal crystal structure. Layers of Sb atoms form a square net. Sheets of Gd atoms are separated by two Te layers. (**b**) Core-level spectrum. Here, we clearly observe sharp peaks due to Te 4*d* (~40 eV), Sb 4*d* (~33 eV) and Gd 4 *f* (~8.5 eV) states. The black dashed line represents the Fermi level. (**c**) Temperature dependence of the reciprocal magnetic susceptibility measured in a magnetic field of 0.5 T applied within the crystallographic a-b plane. Solid line represents the fit of Curie-Weiss law to the experimental data. Upper inset: low-temperature magnetic susceptibility data. Lower inset: magnetic field variation of the magnetization taken at 1.72 K with increasing (full circles) and decreasing (open circles) magnetic field strength. (**d**) *Ab-initio* calculated bulk band structure along the high-symmetry directions. Red circle indicates the approximate position of the Dirac point.

We start our discussion by presenting the crystal structure of GdSbTe. Similar to other ZrSiS-type materials, it has a nonsymmorphic tetragonal unit cell with space group *P4/nmm*. As shown in Fig. 1a, the Gd-Te bilayers are sandwiched between the layers of Sb atoms forming a square net. The single crystals used in the present study were first examined via spectroscopic core-level measurements. The observed sharp peaks (Fig. 1b) due to the Te 4*d* (~40 eV), Sb 4*d* (~33 eV) and Gd 4 *f* (~8.5 eV) electronic states prove the excellent quality of the specimen. Figure 1c displays the magnetic properties of GdSbTe. Above 15 K, the magnetic susceptibility, *χ*(T), obeys a Curie-Weiss law with the effective magnetic moment *μ*_*ef*_ = 7.71 *μ*_*B*_ and the paramagnetic Curie temperature *θ* = −19 K. The experimental value of *μ*_*ef*_ is close to that expected for a trivalent Gd ion (7.94 *μ*_*B*_). The large negative value of *θ* signals strong antiferromagnetic exchange interactions. As shown in the upper inset of Fig. 1c, the compound orders antiferromagnetically at *T*_*N*_ = 13 K, and undergoes another magnetic phase transition near 8 K. At the lowest temperature attained in the present work, i.e. T = 1.72 K, the compound bears an antiferromagnetic state, as supported by the character of the magnetization isotherm displayed in the lower inset of Fig. 1c. The magnetic field variation of the magnetization, *σ*(H), exhibits some tiny inflection near 1.5 T, which can be attributed to a metamagnetic-like phase transition. In stronger fields, *σ*(H) of GdSbTe shows a feebly upward behavior, which indicates that an expected field-induced ferromagnetic arrangement of the gadolinium magnetic moments can be achieved in this compound in magnetic fields much stronger than the maximum field of 5 T available in the present study. The *ab-initio* calculated bulk band structure of GdSbTe is displayed in Fig. 1d. Importantly, at around 0.2 eV below the Fermi energy, a Dirac-like feature with a small gap is observed at the X point of the BZ.

### Fermi surface and observation of nodal-line state

**Figure 2:**
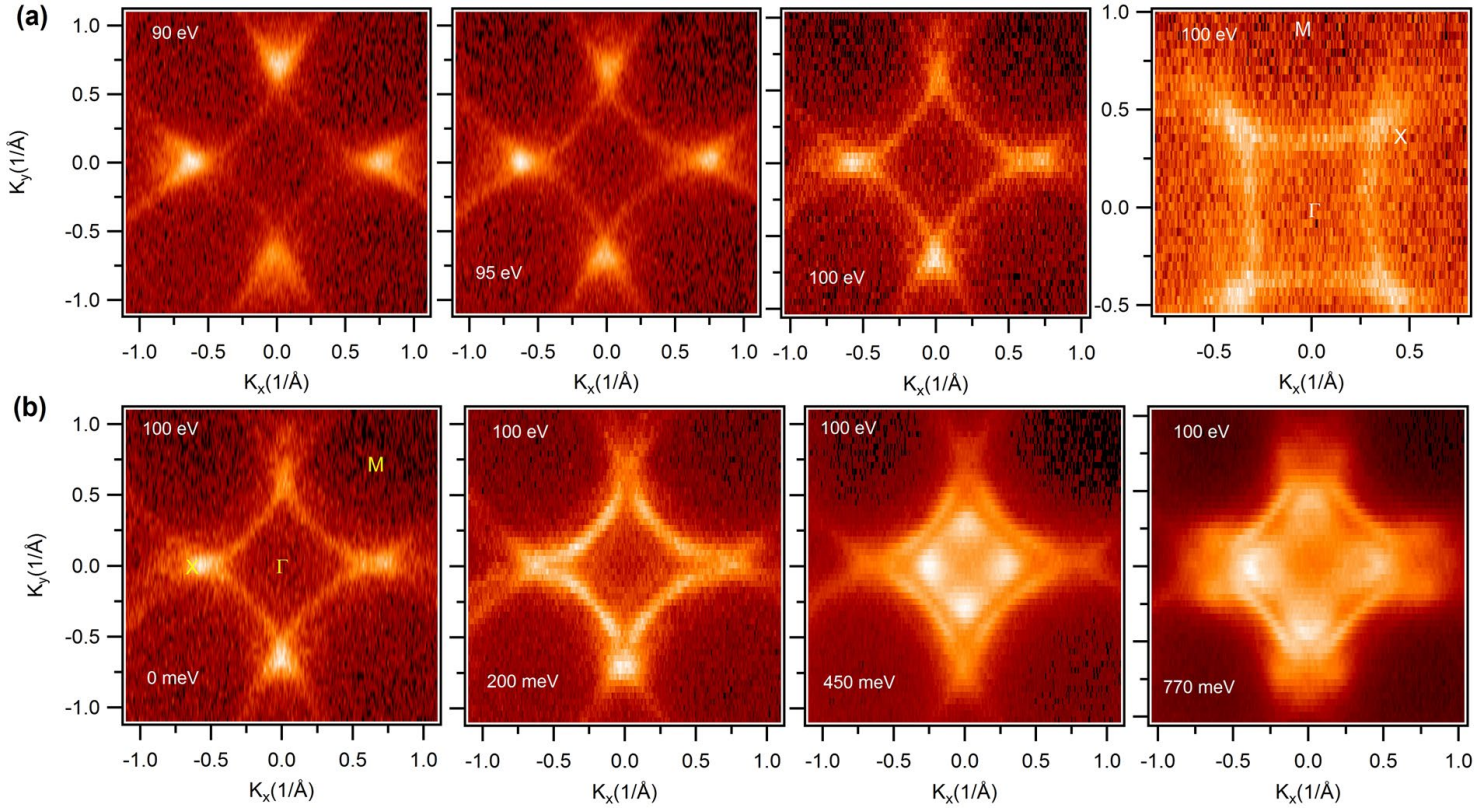
Fermi surface and constant energy contour plots. (**a**) Experimentally measured Fermi surface maps at various photon energies and at different high symmetry directions. The rightmost panel represents the Fermi level in a different orientation with high symmetry points. Photon energies are noted in the plots. (**b**) Constant energy contour plots at various binding energies. Energies are noted in the plots. High symmetry points are indicated in the leftmost plot. All the measurements were performed at the ALS beamline 4.0.3 at a temperature of 21 K.

Figure 2a shows Fermi surface maps of GdSbTe in its paramagnetic states at various incident photon energies. High symmetry points and energies are noted in the plots. The overall Fermi surface is similar to previously reported data on ZrSiS-type materials (see also supplementary information)^30,39^. The topological nodal-line states along the M-Γ-M directions form a diamond shaped Fermi pocket around the center of the BZ. Furthermore, we observe small circular shaped Fermi pockets around the X points of the BZ, respectively (see Supplementary Fig. 3). The rightmost panel of Fig. 2a shows the Fermi surface in a different orientation. Figure 2b shows the constant energy contour plots at various binding energies. Moving towards the higher binding energy we observe that the diamond shape gradually evolves into two diamonds. At a higher binding energy a small circular pocket-like feature evolves along the Γ-X direction. The small pocket is clearly visible in the constant energy contour plot with a binding energy of 770 meV which is in the vicinity of the Dirac point along this direction.

**Figure 3:**
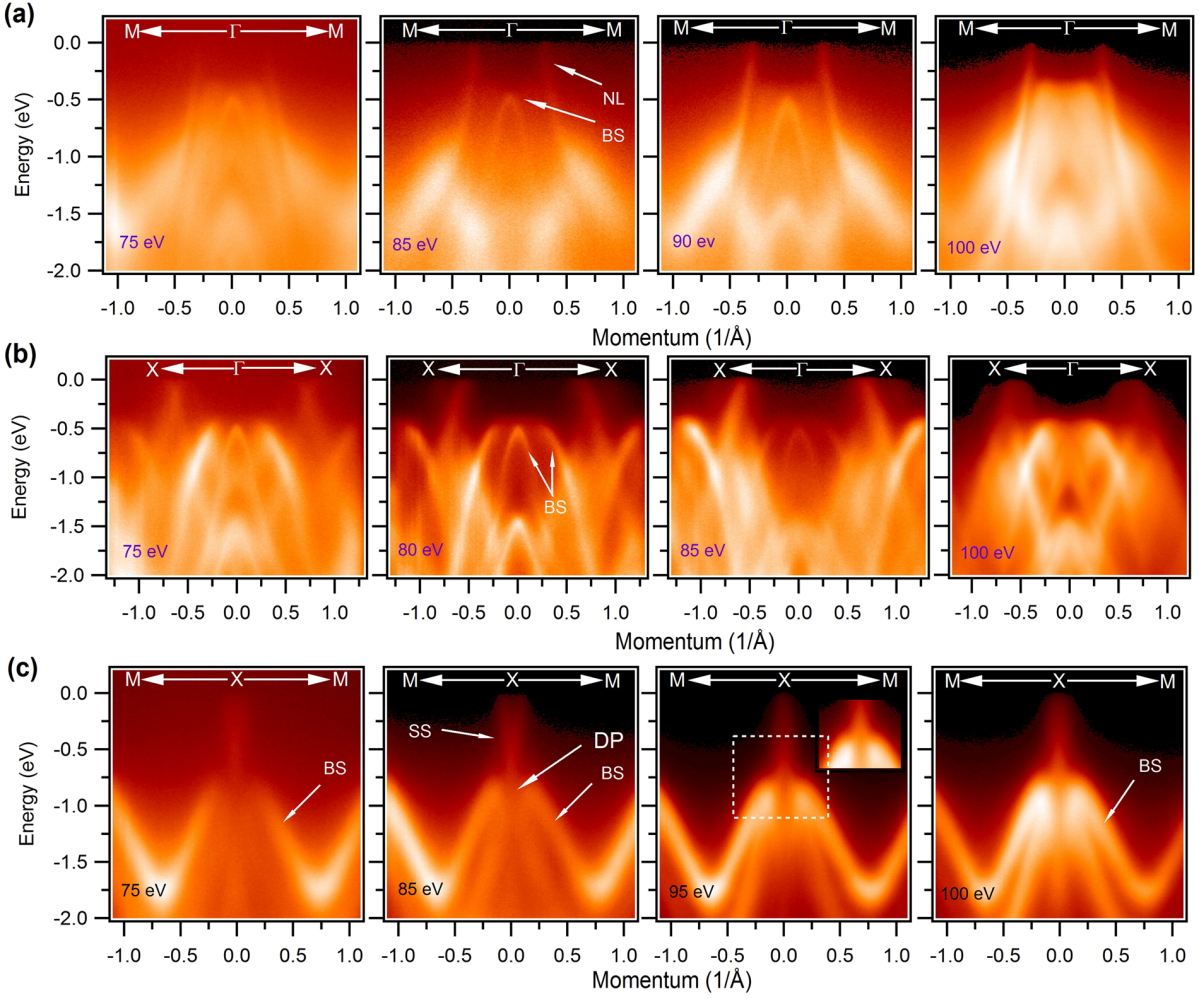
Dispersion map along the high symmetry directions. (**a**) ARPES measured dispersion maps along the M-Γ-M direction at various photon energies. Nodal-line is observed to be in the vicinity of the chemical potential. (**b**) Dispersion maps along the high symmetry X-Γ-X direction. (**c**) Band dispersion along the M-X-M direction. Dirac-like state is observed. Measured photon energies are noted in the plots. Inset shows the zoomed view near the Dirac point marked with dashed white rectangular box. All the measurements were performed at the ALS beamline 4.0.3 at a temperature of 21 K. We note that, NL = nodal line, BS = bulk state, SS = surface state, DP = Dirac point.

**Figure 4:**
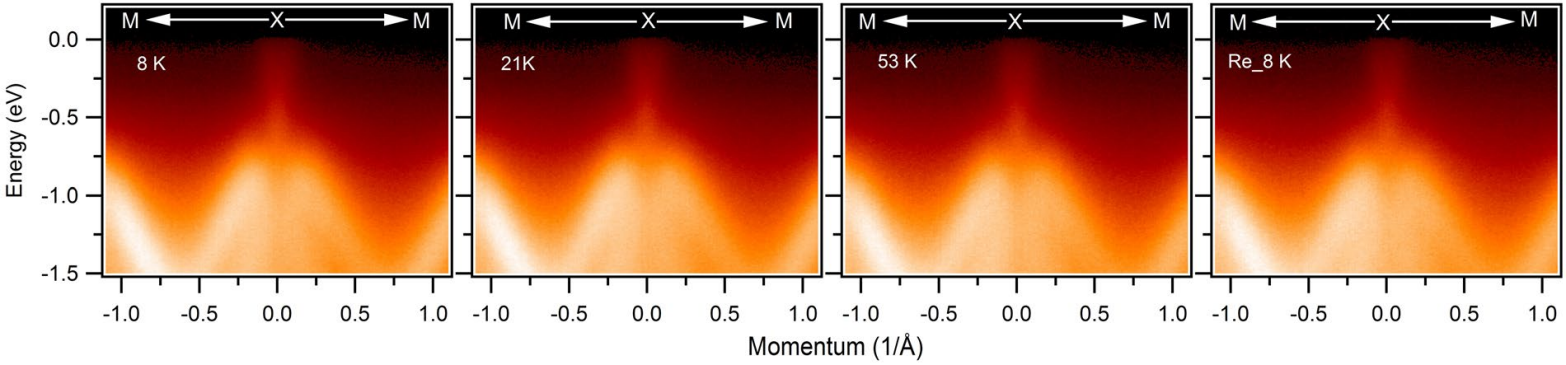
Temperature dependent measurement of dispersion maps along the M-X-M direction. Measured temperature are noted on the plots. Re_8 K indicates the dispersion map after thermal recycle (8 K → 21 K → 53 K → 8 K). All the measurements were performed at the ALS beamline 4.0.3 using a photon energy of 90 eV.

To reveal the nature of the states along the various high symmetry directions, we present photon energy dependent ARPES measured dispersion maps in Fig. 3. The photon energy dependent measurements help to identify the origin of the bands i.e. surface or bulk originated. In contrast to bulk bands, the surface bands do not disperse with incident photon energy. Figure 3a shows the measured dispersion maps along the M-Γ-M direction at various photon energies, where Dirac line node phase has been observed. The photon energies are noted in the plots. The linearly dispersive state which forms the nodal-line (NL) in our measured ARPES spectra do not disperse with the photon energy hence suggesting the surface origin which is consistent with the previously reported result of ZrSiS-type materials^29,30,39^. Furthermore, another bulk band is observed to bend over at around 500 meV below the chemical potential. Figure 3b represents the dispersion maps along the X-Γ-X direction (see Supplementary Fig. 9 and Note 5 for low temperature dispersion maps). A Dirac-like dispersion is observed at around 770 meV below the Fermi level. Near the Γ point two bulk bands are observed where the top of one band is almost flat. Such features are not observed in other reported ZrSiX-type materials, therefore, further theoretical investigation is required. To fully understand the nature of the state at the X point, we present dispersion maps along the M-X-M high symmetry direction in Fig. 3c. Photon energies are noted in the plots. The Dirac like state is observed at the X point of the BZ and does not disperse with photon energy (see also Supplementary Fig. 4 and Note 2). The Dirac point is located at around 770 meV below the Fermi level. Our *ab-initio* slab calculations confirm the existence of this Dirac point near the observed energy, and indicate that this is surface-derived (see Supplementary Fig. 5(b)). In the vicinity of the Dirac point two other bulk bands are observed. This feature is not normal since a surface state usually does not coexist within the projected bulk bands. Therefore, one can expect bulk-surface band hybridization in the vicinity of the Dirac point. Furthermore, we have collected all our data approximately around the normal emission with varying temperature (see Figs. 3c and 4, and Supplementary Figs. 4 and 5) and photon energies where the bands do not show any sort of broadening effect. The inset of Fig. 3(c) shows the zoomed dispersions near the Dirac point where bulk states overlap with the surface state.

### Observation of antiferromagnetic Dirac state

The Dirac semimetal (DSM) conserves the time reversal symmetry and spatial inversion symmetry and requires the protection by some crystalline symmetry^12,13^. The Dirac semimetal breaks into a Weyl semimetal if either of the aforementioned symmetries is broken. Interestingly, the Dirac semimetal may exist even if both time reversal symmetry and inversion symmetry are broken^23,24^. This kind of situation is proposed to exist in some antiferromagnetic semi-metallic materials. When a material undergoes a magnetic phase transition from paramagnetic to ferromagnetic or antiferromagnetic, the time reversal symmetry is broken. If time reversal symmetry (*T*) and spatial inversion symmetry (*P*) are broken, the Dirac semimetal no longer exists, however, their product may remain conserved^24^. For the DSM to survive, the desired AFM material should exhibit ordered moments such that *P* and *T* are individually broken but *PT* is conserved and in addition the crystalline symmetry (in the case of the 111 compounds the non-symmorphic symmetry) protecting the DSM state in the non-magnetic state is not violated. In addition, the AFM state would need to be robust against SOC and correlation effects so that the aforementioned conditions can still be met^24,28^. Since the antiferromagnetic (AFM) transition temperature is 13 K in the GdSbTe system, it can provide a platform to study the effect of magnetism and topology. We present dispersion maps along the M-X-M direction both below and above the magnetic transition temperature of this material in Fig. 4. Temperature values are indicated in the plots. Interestingly, we observe robust Dirac like state in both paramagnetic and antiferromagnetic phases (see also Supplementary Fig. 5 and Note 3). Our thermally recycled dispersion maps (8 K → 21 K → 53 K → 8 K) indicates the robust nature of the Dirac state in GdSbTe. Although the time reversal symmetry (*T*) is broken in the magnetic phase of GdSbTe, a potential product of the rotation-inversion operator with the time-reversal operator may be responsible for topological state protection in the AFM state. Furthermore, owing to the robust Dirac state at the X point of the BZ, one can expect to see the effect of magnetism at the other points of the BZ. To show that, we have performed spectral weight transfer analysis and symmetrization of the energy distribution curves along the nodal-line direction (see Supplementary Note 4 and Figs 7 and 8). Clear observation of a large deviance of intensities between the energy range from −0.15 to 0 eV and a kink in the symmetrized data confirm the effect of magnetism in the nodal-line direction.

## Discussion

**Figure 5:**
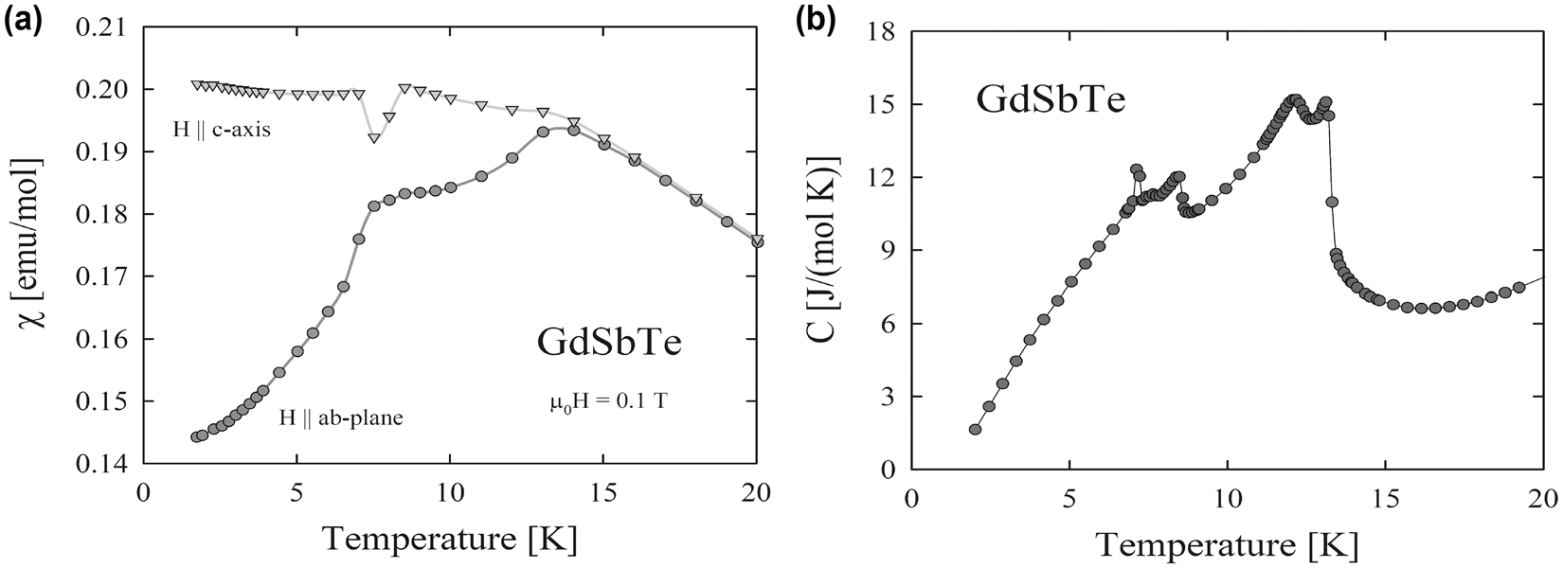
Low-temperature thermodynamic properties of single-crystalline GdSbTe. (**a**) Temperature dependence of the magnetic susceptibility measured in a magnetic field of 0.1 T applied along the crystallographic *c* axis (triangles) and the tetragonal *a*–*b* plane (circles). (**b**) Temperature dependence of the specific heat.

In order to shed some light on the magnetic structure of GdSbTe and the corresponding topological states below *T*_*N*_, low-temperature measurements of the thermodynamic properties were performed (see Fig. 5). It should be noted that the strongly uniaxial crystal structure of this compound favors an Ising type of spin arrangement that is indeed observed for numerous isostructural antiferromagnets. Usually, the magnetic structures of such phases consists of ferromagnetically coupled (001) planes which are arranged along the *c*-axis in an antiferromagnetic +−, ++ −−, or +− −+ manner. For these types of magnetic ordering a spatial rotation-inversion operator might combine with time-reversal operator to provide the necessary preservation of symmetry that enables the protection of the topological states^23^.

However, the magnetism in GdSbTe is complex and not yet resolved. Our magnetic data (see Fig. 5a) indicate that in the ordered state the Gd magnetic moments are confined within the tetragonal *a–b* plane. The magnetic susceptibility measured along and perpendicular to the *c* axis exhibits in the ordered state quite unusual temperature dependencies, which hints the occurrence of several spin reorientation transitions. This finding is supported by the heat capacity data (see Fig. 5b). Determination of the spatial inversion operator, or perhaps a complex yet expected for this structure roto-inversion operator will be possible once the multiple magnetic structures in GdSbTe are determined.

In conclusion, we have performed an experimental study of GdSbTe using ARPES and thermodynamic measurements. The results were supplemented by first-principles calculations. Our studies revealed the topological nodal state and confirmed that the overall electronic structure in GdSbTe is similar to those observed for ZrSiS-type materials. Most importantly, we discover a Dirac-like state both below and above the magnetic transition temperature of 13 K. With a relatively high Néel temperature, GdSbTe is a good platform for studying the interplay between magnetism and topology.

## Methods

### Crystal growth and characterization

Single crystals of GdSbTe were grown by chemical vapor transport method using iodine as a transporting agent^42,43^. The chemical composition of the crystals was checked by energy-dispersive X-ray analysis using a FEI scanning electron microscope equipped with an EDAX Genesis XM4 spectrometer. Single crystal X-ray diffraction (XRD) experiment was performed on a Kuma-Diffraction KM4 four-circle diffractometer equipped with a CCD camera using Mo K*α* radiation. The results indicated the tetragonal space group *P4/nmm* (No. 129) with the lattice parameters a = 4.3104(5) Å and c = 9.1233(15) Å. The XRD scans revealed that the platelet-shaped crystals have their large surface perpendicular to the crystallographic c-axis. Magnetic measurements were performed in the temperature range from 1.72 K to 400 K and in external fields up to 5 T using a Quantum Design MPMS SQUID magnetometer. The heat capacity was measured in the temperature range 2–20 K employing a Quantum Design PPMS platform.

### Spectroscopic characterization

Synchrotron based ARPES measurements were performed at Advanced Light Source (ALS) beamlines 4.0.3 and 10.0.1.1 equipped with a Scienta R8000 and R4000 hemispherical electron analyzer setup, respectively. Similarly, helium lamp based ARPES measurements were performed at the Laboratory for Advanced Spectroscopic Characterization of Quantum Materials (LASCQM) with R3000 hemispherical analyzer at University of Central Florida. The angular resolution was set to be better than 0.2°. And the energy resolution was set to be better than 20 meV. The samples were cleaved *in-situ* under the vacuum condition better than 3 × 10^−11^ torr and at a temperature around 21 K.

### Electronic structure calculations

The electronic structure calculations were carried out using the Vienna *ab-initio* Simulation Package (VASP)^44,45^, and the generalized gradient approximation (GGA) used as the DFT exchange-correlation functional^46^. Projector augmented-wave pseudopotentials^47^ were used with an energy cutoff of 500 eV for the plane-wave basis, which was sufficient to converge the total energy for a given *k*-point sampling. To simulate the surface effects, we used 1 × 5 × 1 supercell for the (001) surface, with a vacuum thickness larger than 19 Å.

## Electronic supplementary material

Supplementary Information

## Data Availability

The data that support the findings of this study are available from the corresponding author upon request.
