# Supplementary material for: Discovery of topological nodal-line fermionic phase in a magnetic material GdSbTe
Source: Sci Rep. 2018 Sep 5;8:13283. doi: 10.1038/s41598-018-31296-7 (PMC6125290; doi:10.1038/s41598-018-31296-7)
Supplement: Supplementary file 1 — Supplementary Information [file 41598_2018_31296_MOESM1_ESM.pdf]

Supplementary Information

Discovery of topological nodal-line fermionic phase in a magnetic material GdSbTe

M. Mofazzel Hosen[1], Gyanendra Dhakal[1], Klauss Dimitri[1], Pablo Maldonado[2], Alex Aperis[2], Firoza Kabir[1], Christopher Sims[1], Peter Riseborough[3], Peter M. Oppeneer[2], Dariusz Kaczorowski[4], Tomasz Durakiewicz[5,6], Madhab Neupane[1]

August 1, 2018

[1]Department of Physics, University of Central Florida, Orlando, Florida 32816, USA

[2]Department of Physics and Astronomy, Uppsala University, P. O. Box 516, S-75120 Uppsala, Sweden

[3]Department of Physics, Temple University, Philadelphia, PA 19122, USA

[4]Institute of Low Temperature and Structure Research, Polish Academy of Sciences, 50-950 Wroclaw, Poland

[5]Condensed Matter and Magnet Science Group, Los Alamos National Laboratory, Los Alamos, NM 87545, USA

[6]Institute of Physics, Maria Curie-Sklodowska University, 20-031 Lublin, Poland

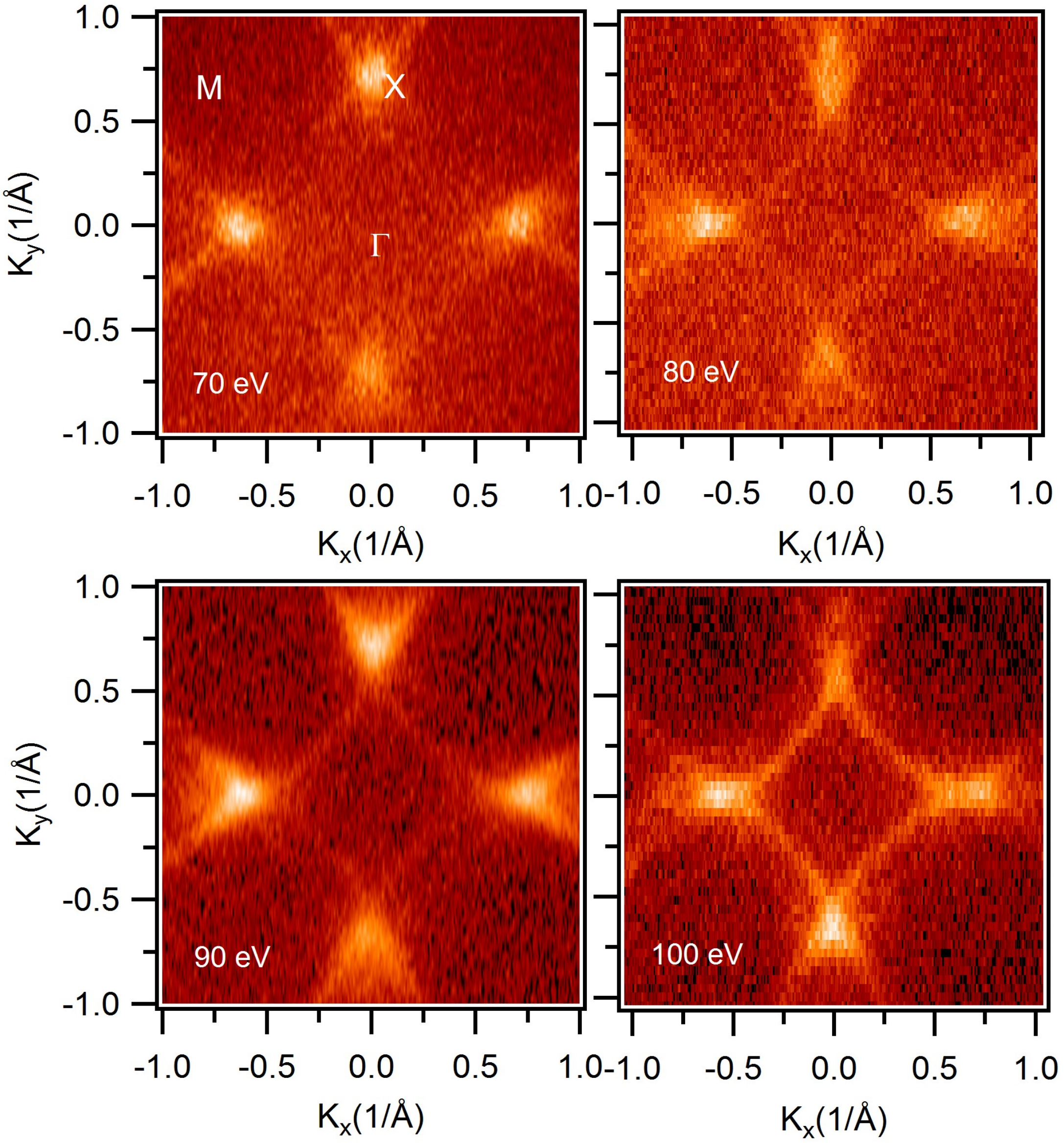

Supplementary figure 1: **Fermi surface maps of GdSbTe.** Fermi surface maps at different photon energies. Photon energies are marked in the plots. All the measurements were performed at ALS beamline 4.0.3 at a temperature of 21 K.

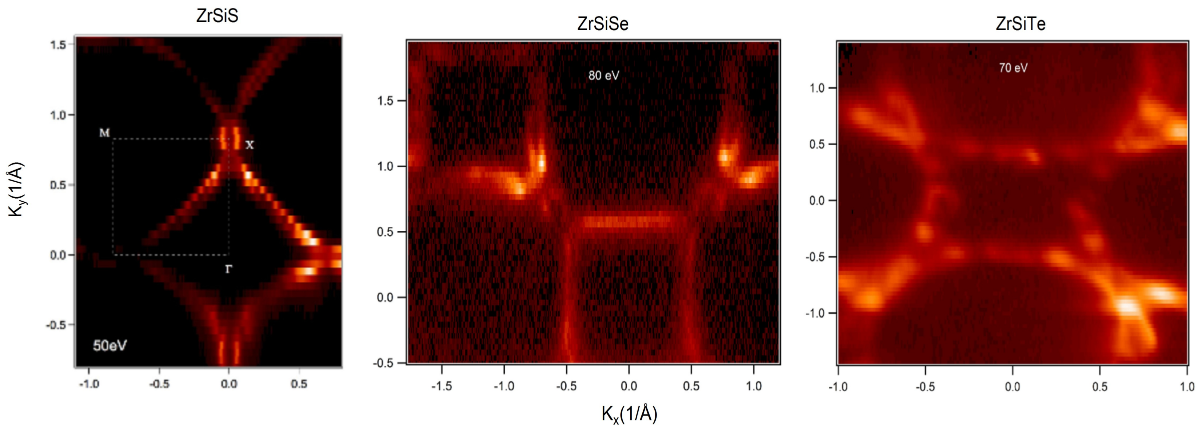

Supplementary figure 2: **Fermi surface maps of other members of 111 material.** Fermi surface maps of ZrSiS, ZrSiSe, and ZrSiTe [1, 2]. Photon energies and high symmetry points are marked in the plots. A Diamond-shaped pocket at the zone center of the Brillouin zone is observed in all three materials.

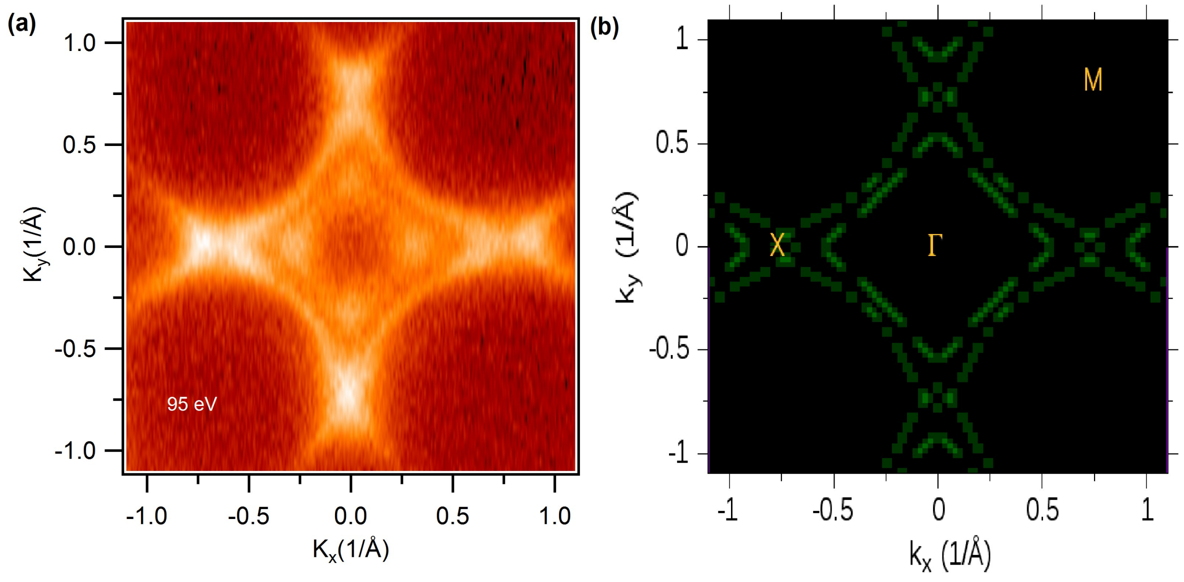

Supplementary figure 3: **Constant energy contour plots of GdSbTe. (a)** Constant energy map slightly below the chemical potential measured at a temperature of 21 K using a photon energy of 95 eV. **(b)** Calculated constant energy contour plot slightly above the chemical potential. The calculations were performed using DFT + U method. High symmetry points are noted in the plot.

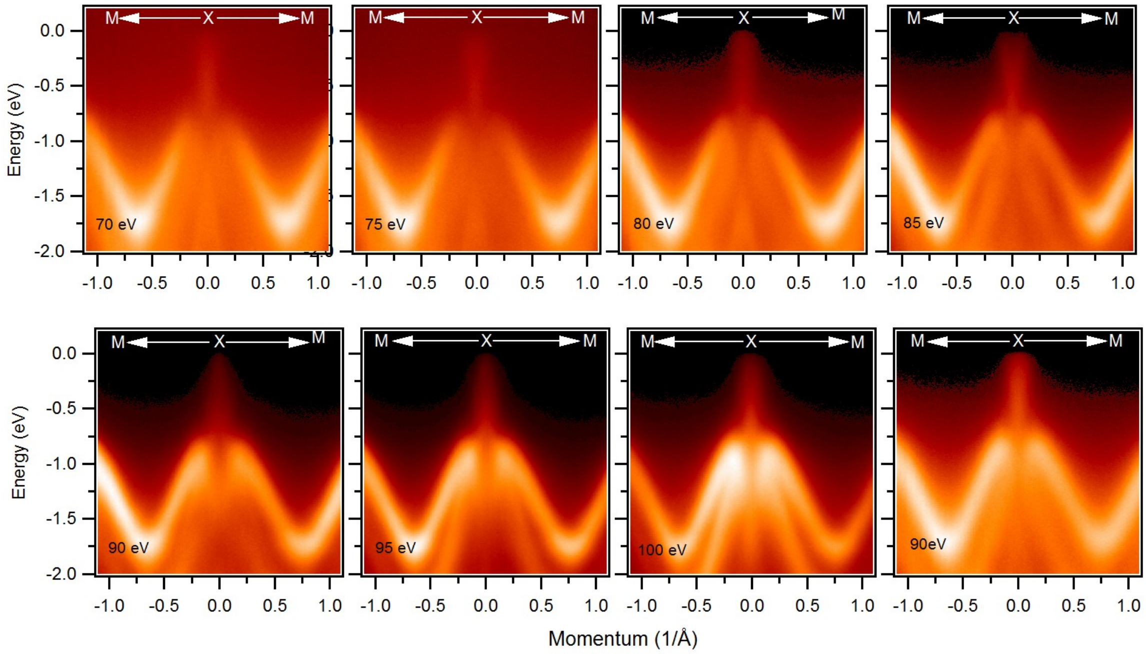

Supplementary figure 4: **Observation of Dirac state.** Photon energy dependent dispersion maps measured at 21 K with photon energies from 70 eV to 105 eV with every 5 eV energy steps. From these plots it is clear that the Dirac state does not disperse with the photon energy i.e, this asserts Dirac state is indeed a surface state.

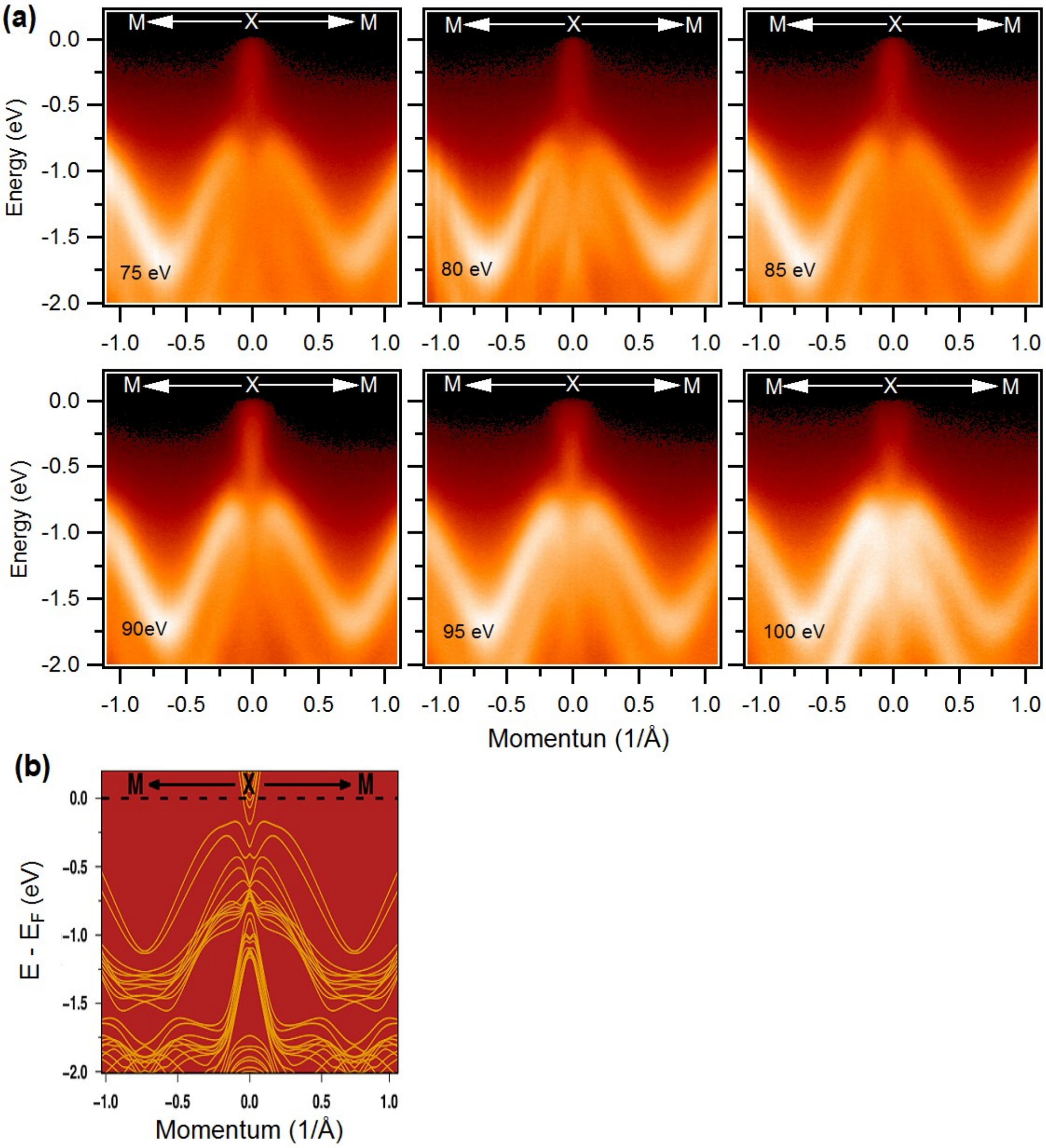

Supplementary figure 5: **Low temperature dispersion map along the M-X-M direction. (a)** Photon energy dependent dispersion maps measured at a temperature of 8 K which is below the magnetic transition temperature (13 K). Photon energies are indicated in the plots. Here, it is clearly observed that the Dirac state does not disperse with the photon energy. **(b)** *Ab initio* calculated band structure of magnetic GdSbTe along the symmetry line M-X-M for a slab geometry. The gapless Dirac state is found around 770 meV below the Fermi level at the X point, in good agreement with experiment. The calculated bands forming the Dirac point are derived from the surface of the slab.

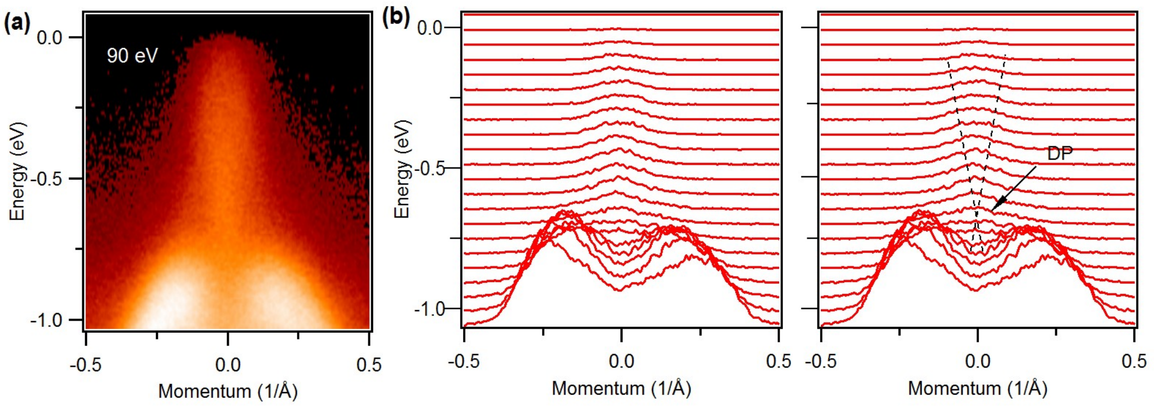

Supplementary figure 6: **Observation of Dirac point at the X point of the BZ.** **(a)** Measured dispersion map at the X point of the BZ using a photon energy of 90 eV. **(b)** MDCs of the same plot. Dashed line in the right panel serves as guide to the eyes.

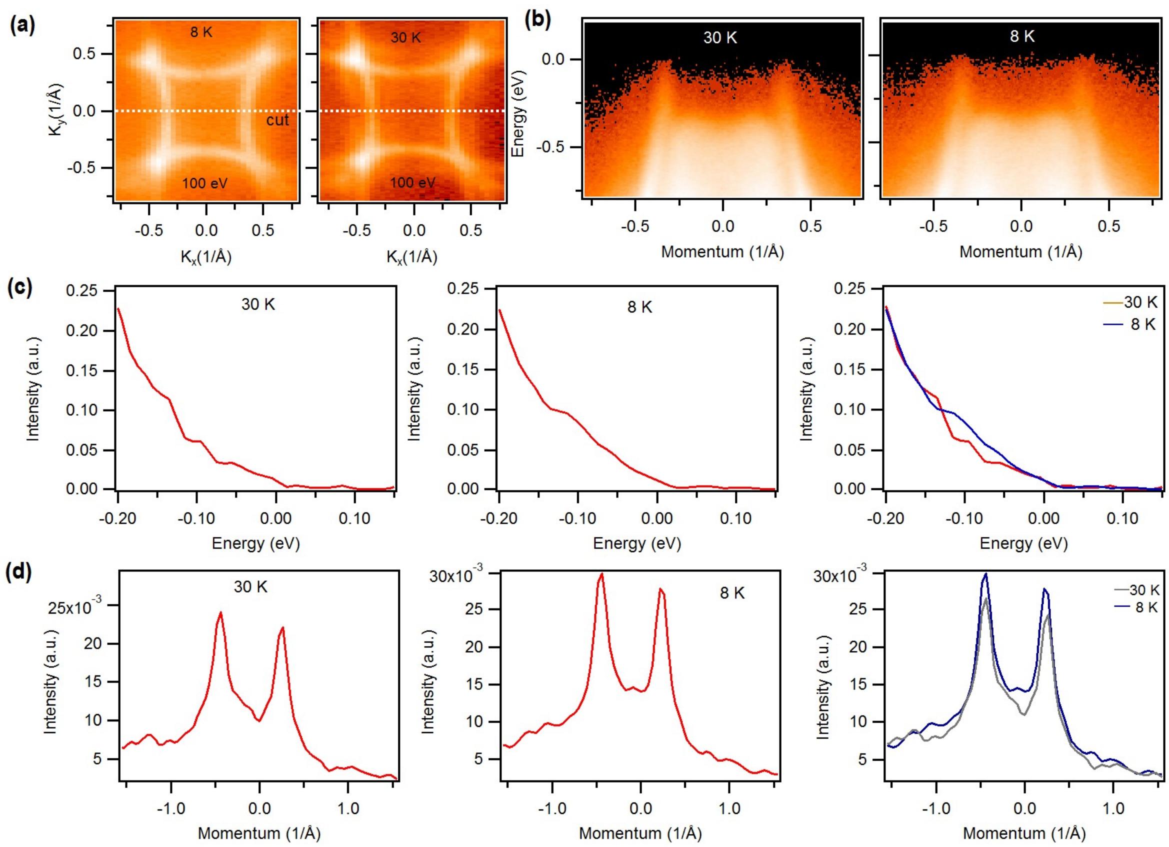

Supplementary figure 7: Effect of magnetism along the nodal-line directions. **(a)** Measured Fermi surface both below and above the magnetic transition temperature. The white dashed line represents the cut direction along the nodal-line. **(b)** Dispersion maps along the nodal-line direction taken at 30 K and 8 K, respectively. **(c)**,**(d)** Momentum and energy distribution curves at paramagnetic phase (left), antiferromagnetic phase (middle) and their comparison. The temperature values are noted in the plots.

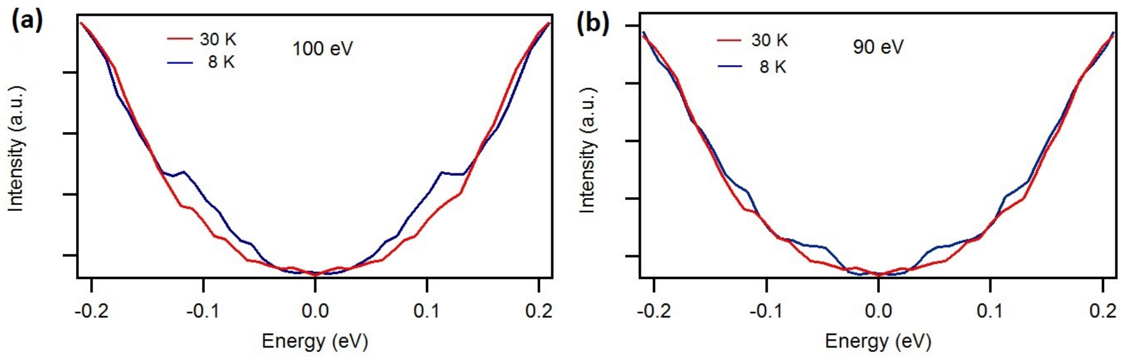

Supplementary figure 8: **Symmetrized EDC plot. (a)**,**(b)** Symmetrized EDC plot of the raw data at both antiferromagnetic (blue) and paramagnetic phase (red) measured at a photon energy of 100 eV and 90 eV, respectively.

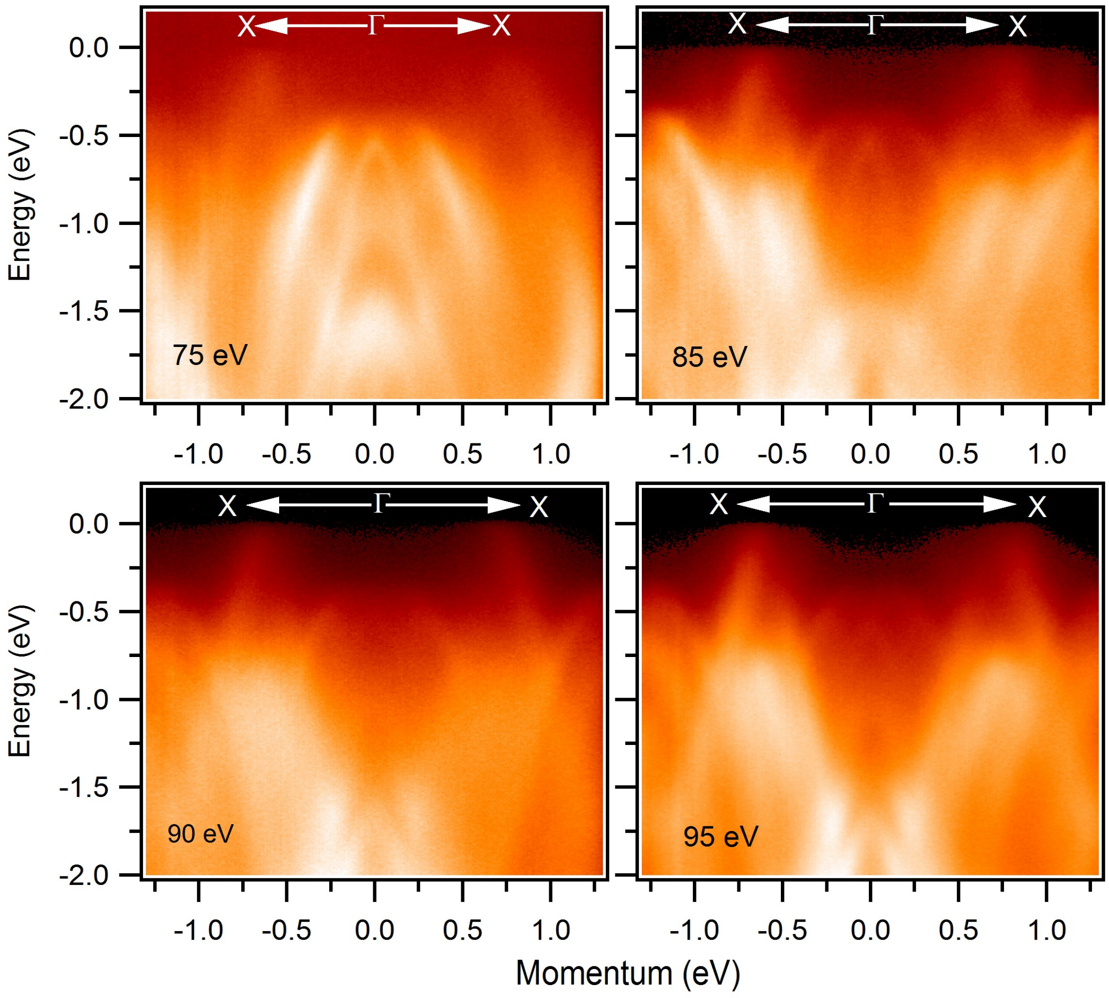

Supplementary figure 9: **Low temperature dispersion maps along X-Γ-X direction.** Photon energy dependent dispersion maps along the X-Γ-X direction measured below the magnetic transition temperature (13 K). Photon energies are noted in the plots.

Supplementary note 1: Comparison of Fermi surface maps of GdSbTe with ZrSiS-type 111 materials. In order to compare our measured electronic structure of GdSbTe with ZrSiS-type of 111 materials, we present Fermi surface maps of GdSbTe, ZrSiS, ZrSiSe, and ZrSiTe [1, 2] in Supplementary Figures 1 and 2. Supplementary Figure 1 shows the measured Fermi surface maps of GdSbTe at various photon energies ranging from 70 eV to 100 eV. Figure 2 represents the Fermi surface maps of ZrSiS, ZrSiSe, and ZrSiTe, respectively. By comparing both figures we can clearly observe all the main features such as a diamond-shaped pocket at the zone center Brillouin zone (BZ). Furthermore, we carried out DFT + U calculations which we compare with our experimental observations. Supplementary Figure 3 shows the measured and calculated constant energy contour plots of GdSbTe. Our calculated energy contour plot reproduces all the major features as shown in supplementary Figure 3a,b. Therefore, we conclude that the overall electronic structure of GdSbTe agrees with the other members of the 111 material group.

Supplementary note 2: Observation of robust Dirac state at the X-point of the BZ. The photon energy dependent measurements can unambiguously confirm the origin of bands i.e. either it is surface or bulk originated. With photon energy, surface originated bands should not disperse while this argument is not true for bulk originated bands. In Supplementary Figure 4, we present the dispersion maps along the M-X-M direction using a wide energy range from 70 eV to 105 eV. Within this wide energy range, the Dirac-like linearly dispersive state does not disperse. Therefore, we attribute these bands as surface originated. Furthermore, with temperature dependent measurements in the main text of Fig. 4, we confirm that the linearly dispersive state at the X-point is robust in nature.

Supplementary note 3: Observation of robust Dirac point at antiferromagnetic phase. We present photon energy dependent dispersion maps at low temperature (8 K) along the M-X-M direction of GdSbTe in Supplementary Figure 5(a). This will help us to confirm the nature of the bands along this high symmetry direction in the antiferromagnetic phase of this material. We can clearly observe that the Dirac states do not show any dispersion with the changing photon energy. Supplementary Fig. 5(b) represents the *Ab initio* calculated band structure of magnetic

GdSbTe along the symmetry line M-X-M for a slab geometry. Gapless Dirac state is observed at around 770 meV below the Fermi level (see also Supp. Fig. 6). Therefore, this further corroborates our main text conclusion of a robust surface originated Dirac state in the antiferromagnetic phase of this material.

Supplementary note 4: Observation of the effect of magnetism along the nodal-line. From supplemtary Fig. 4 and 5, we have seen that Dirac node at the X point is robust against the magnetism. Therefore, we have performed detailed spectral weight distribution analysis along the nodal-line direction in order to observe the effect of magnetism. Supplementary Figure 7(a) shows the Fermi surface maps measured with 100 eV photon energy at the paramagnetic (right) and antiferromagnetic phase (left). The white dashed line represents the cut directions presented in Supplementary Fig. 7(b). Supplementary Figure 7(c),(d) show the MDC and EDCs along the nodal-line at both magnetic phases of GdSbTe. Here, we observe change of specral weight in the antiferromagnetic state of GdSbTe. It is possible that MDC peak can slightly enhanced by going to the lower temperature, however the spectral weight transfer kink at EDC confirms that its not due to the temperature as the enhancement should happened uniformly. Therefore, observation of kink at the 0.1 eV below the chemical potential shows the signature of magnetism along this direction. Additionally, on the MDC curves of 30 K and 8 K measurements, we have observed significantly notable dichotomy at the X point, where the derivatives of the 30 K measurement reads a local minima but the 8 K measurement reads a small local maxima. To subtract the temperature broadening effect, we have symmetrized the ARPES spectra (EDC) along the nodal node (see supplementary Fig. 8). Here, once again we clearly observe the kink i.e, we can conclude this spectral weight transition as a signature of the magnetism, not just due to thermal broadening.

Supplementary note 5: Low temperature dispersion maps along the X-Γ-X direction. To compare the band structure along the high symmetry points both above and below the transition temperature, we present dispersion maps along the X-Γ-X direction (see Supplementary Figure 9). Comparing with Fig. 3b in the main text, we observe that the overall band dispersion agrees well above and below the magnetic transition temperaure. These dispersions are measured at 8 K which is

below the magnetic transition temperature (13 K).

References

[1] Neupane, M. *et al.* Observation of Topological Nodal Fermion Semimetal Phase in ZrSiS. *Phys. Rev. B* **93**, 201104(R) (2016).

[2] Hosen, M. M. *et al.*, Tunability of the topological nodal-line semimetal phase in ZrSiX-type materials. *Phys. Rev. B* **95**, 161101(R) (2017).
